# The Alliance Hypothesis for Human Friendship

**DOI:** 10.1371/journal.pone.0005802

**Published:** 2009-06-03

**Authors:** Peter DeScioli, Robert Kurzban

**Affiliations:** University of Pennsylvania, Philadelphia, Pennsylvania, United States of America; Harvard University, United States of America

## Abstract

**Background:**

Exploration of the cognitive systems underlying human friendship will be advanced by identifying the evolved functions these systems perform. Here we propose that human friendship is caused, in part, by cognitive mechanisms designed to assemble support groups for potential conflicts. We use game theory to identify computations about friends that can increase performance in multi-agent conflicts. This analysis suggests that people would benefit from: 1) ranking friends, 2) hiding friend-ranking, and 3) ranking friends according to their own position in partners' rankings. These possible tactics motivate the hypotheses that people possess *egocentric* and *allocentric* representations of the social world, that people are motivated to conceal this information, and that egocentric friend-ranking is determined by allocentric representations of partners' friend-rankings (more than others' traits).

**Methodology/Principal Findings:**

We report results from three studies that confirm predictions derived from the alliance hypothesis. Our main empirical finding, replicated in three studies, was that people's rankings of their ten closest friends were predicted by their own perceived rank among their partners' other friends. This relationship remained strong after controlling for a variety of factors such as perceived similarity, familiarity, and benefits.

**Conclusions/Significance:**

Our results suggest that the alliance hypothesis merits further attention as a candidate explanation for human friendship.

## Introduction

Although friendship is a core element of human social life, its evolved functions remain poorly understood [1]. Human friendship often occurs among individuals who are neither relatives nor mates, ruling out key explanations for cooperation such as kin selection. Nonetheless, similar relationships have been observed in non-human species, and understanding of these long-term, dyadic, non-kin, non-sexual relationships has progressed apace. Hyenas use partners to gain access to carcasses [2], male dolphins employ partners to attain females for mating [3], juvenile rooks use partners to get food [4], and numerous primate species groom partners to garner agonistic support [5]. From a functional perspective, to the extent that an organism is designed to influence other individuals, these individuals can be understood as devices in the organism's “extended phenotype” [6]. What are the evolved functions of human friends?

Traditional evolutionary approaches explain human friendship by applying the theory of reciprocal altruism [7]. On this view, friends function as exchange partners, from whom gains in trade can be profitably extracted, provided that cheaters can be avoided. However, a wealth of empirical evidence from social psychology is inconsistent with the exchange theory. Contradicting a key prediction of reciprocity theories, people do not carefully monitor benefits given and received in close relationships [1], [8]–[12]. Also, people seem to help friends even when they are unlikely to be capable of repayment [12]. This suggests that friendship involves more than exchange.

Friendship might be illuminated by considering other cognitive systems, in addition to exchange mechanisms, that humans use to manage the complex social world [13], [14]. Specifically, we consider this hypothesis: Friendship is generated, in part, by cognitive systems that function to assemble a support group for potential conflicts. This “alliance hypothesis” proposes that human friendship is less like trade and more like alliance politics. Human conflicts are usually decided by the number of supporters mobilized on each side (rather than strength or agility). This is true for a wide range of disputes, from family debates over weekend plans [15] to homicidal attacks [16]. Therefore, individuals can increase their power by creating and maintaining a network of allies, well in advance, before the onset of an argument or quarrel.

Here we develop and test predictions derived from the alliance hypothesis. One central prediction is that alliance-building mechanisms should evaluate partners' loyalties to their other friends, using this information to rank friends according to how they rank the self. Our main empirical finding, replicated in three studies, was that people's rankings of their ten closest friends were predicted by their own perceived rank among their partners' other friends. This relationship remained strong after controlling for a variety of factors such as perceived similarity, familiarity, and benefits. These results suggest that a new variable–perceived rank–plays a crucial role in friendship and that the alliance hypothesis merits further attention.

### The Puzzle of Communal Relationships Among Nonkin

Clark and Mills [9] distinguished between exchange relationships, in which individuals give benefits and expect repayment, and communal relationships, in which individuals give benefits according to the recipient's needs, without expecting a specific response. They found that people seeking an exchange relationship preferred partners who returned favors, whereas people seeking a communal relationship showed greater liking for partners who did not give benefits in return. In a subsequent experiment [8], pairs of participants (strangers or friends) completed a joint task for a reward which could be divided equally or according to their respective contributions. Pairs of strangers typically tracked individual contributions by using different color pens, while friends generally chose not to monitor inputs. A similar experiment showed that strangers frequently monitored a light that indicated their partner's contributions whereas friends did so much less often [17].

Similarly, Fiske's [10] relational models theory asserted that exchange and communal relationships are fundamentally distinct, comprising two of the four basic psychological models used to manage human social life. Fiske claimed that communal sharing relationships are based on a “principle of equivalence” that facilitates sharing and “makes it impossible to make graduated differentiations among people” (p. 716). The exchange/communal distinction is supported by a diverse array of evidence, including ethnographic fieldwork [18] and a series of experiments showing that relationship type explains how people categorize [19], recall [20], substitute [21], and misidentify [22], [23] individuals with whom they have relationships. Fiske's theory has also been useful for understanding taboo thinking [11] and indirect speech [24].

Taken together, the empirical evidence shows that close relationships are conceptualized and structured in a way that fundamentally differs from exchange relationships. How, then, did the cognitive systems underlying communal relationships evolve? Researchers in this area typically appeal to some form of kin selection, such as the evolution of parental care mechanisms [10], [25]. Kin selection might explain communal relationships among relatives, but what explains communal relationships among nonkin? It is sometimes assumed that friendships are caused by mistakes in systems designed for kin altruism. However, as Silk [1] has pointed out, this idea is implausible, as it implies that humans are less flexible and discriminating in relationships than nonhuman primates. Another idea is that people form committed friendships to buffer against potential crises such as illness or injury; in this model, people commit to a friend to solicit the friend's commitment because faking commitment is difficult [12].

In sum, friendship remains puzzling. What evolved functions are performed by the cognitive systems underlying communal friendships?

### Alliance Mechanics

The alliance hypothesis is derived from ideas developed in game theory [26] and international relations [27]. Just as aerodynamic theory can describe a gradient of functionality for flight mechanisms, game theory can describe a gradient of functionality for strategic decision-making, thereby allowing performance-enhancing cognitive mechanisms to be identified.

Alliance formation fundamentally differs from reciprocal exchange in important respects. Whereas exchange can be modeled with two-player games (like the Prisoner's Dilemma), alliances are defined in games with three or more players [26, see pp. 220–237]. The simplest alliance problem is a zero-sum three-player game like the Simple Majority Game [26] in which three players each choose one of the other players. If two players choose each other, they form an alliance and get ½ each, while the excluded player gets -1; if no two players choose each other, all players get 0. Beyond this simplest case, more complex alliance problems can be described by adding asymmetric alliance strengths, within-alliance bargaining, nonzero-sum payoffs, more players, more strategies, uncertainty, and so on [26].

The Simple Majority Game shows that alliance formation, even in the simplest case, is qualitatively different from two-player exchange because in alliance formation, *benefiting one player requires harming another* [26]. George Liska, in his landmark treatise on alliances, wrote that “alliances are against, and only derivatively for, someone or something” [28, p. 12]. In these situations, exchange strategies like tit-for-tat [29] are unworkable: When there is a dispute between two of an individuals' cooperative partners (both with high probabilities of repetition and histories of cooperation), choosing sides makes it impossible to match cooperation with cooperation for both partners.

We are not suggesting, of course, that in all multi-player conflicts, individuals must necessarily choose sides, i.e., form alliances. People apply a wide breadth of tactics to manage others' conflicts such as mediation, arbitration, and suppression [30]. We simply point out that when disputants are unable to reconcile their interests, then third parties face the unique and difficult problem of deciding to help/harm one side or the other.

In short, the core problem in alliance decisions is choosing sides. There is no consensus on the correct normative model for these choices. However, we highlight two decision procedures drawn from the international relations literature [27].

One decision procedure is *bandwagoning* [27]. Individuals can side with the disputant that seems most likely to win the argument or quarrel. This method helps individuals avoid ending up on the losing side of a fight–a potentially costly outcome. Any initial advantage by one disputant is magnified when third parties engage in bandwagoning. Individuals with more supporters are more likely to win, and they get more supporters as a result, setting in motion a self-reinforcing process in which the powerful get more powerful.

Alternatively, individuals can side with the disputant who would be most likely to side with them in potential future conflicts. By protecting their own supporters, individuals can increase their future power to prevail in conflicts. This decision procedure creates feedback loops of affinity among allies. When an individual shows allegiance to their partner, they become more valuable to that partner, who in turn increases allegiance to the individual, and so on. The escalating affinity between Britain and France in the decade leading into the First World War provides an illustrative example [31]. This feedback dynamic can be described as an “integrative spiral” [31] or as *alliance-building* (the reverse occurs among adversaries). The result is that an individual can come to deeply value a partner *precisely because they are allies*, independent of the partner's desirable or undesirable traits.

Bandwagoning and alliance-building are opposing forces that can result in a wide range of group coalition structures depending on their relative strengths [32]. Bandwagoning pushes group structure toward a linear dominance hierarchy with one extremely powerful individual who leverages unanimous group support. Alliance-building pulls group structure toward pairs who loyally defend each other. A variety of intermediate alliance structures exist between these extremes.

Finally, the tactics described above imply that alliance information is very sensitive. Third parties might take one side or another depending on their true or false knowledge of others' allegiances. Thus individuals should display, conceal, or distort information about their alliances as required. As Snyder [31] has shown, one important case is a “straddle strategy” in which individuals conceal their allegiances. Low-valued partners might not shift loyalties if they don't know their low value; high-valued partners might be less emboldened to provoke a fight if alliance support is uncertain.

### Do Friends Function as Allies?

Alliances and exchange pose different adaptive problems which require different information-processing solutions. Researchers have identified a number of computations that support successful exchange [7], [29], including individual recognition, memory of transaction histories, and the ability to detect and discriminate against non-reciprocators. What cognitive mechanisms might help individuals navigate the world of alliances?

Multi-player conflicts are complex and require sophisticated computations. To accomplish bandwagoning, individuals must estimate the relative power of any two disputants, which might depend on many factors including their respective alliances. For example, hyenas maintain representations of all group members' statuses, and they use status information to choose sides in conflicts, always siding with the higher status fighter [33].

Siding with one's more reliable allies is even more cognitively demanding. Individuals need an *egocentric* representation of other group members indicating which side to take for any given conflict. The simplest specification of this type would be a transitive *ranking* of other group members. To protect their more reliable allies, individuals should rank partners according to how their partners rank them, preferring partners who rank them higher. To accomplish this, individuals need representations of other group members' loyalties to the other group members. That is, they must represent the world of alliances as it is seen by everyone else. Borrowing terminology from the spatial cognition literature [34], individuals need to maintain *allocentric* representations of the social world.

Thus, the adaptive problems posed by alliance and exchange differ in their computational descriptions. These candidate functions therefore make different predictions about the cognitive processes underlying friendship. Most basically, each type of system should seek different information. Exchange mechanisms should focus on one's own transaction histories and expectations about future interactions; bandwagoning mechanisms should assess others' power and parse status hierarchies; and alliance-building mechanisms should evaluate others' relative loyalties to other group members. By examining the mental processes underlying friendship, it might be possible to distinguish the functions these systems perform.

### The Present Studies

An obvious first step toward understanding friend cognition is to examine the direct product of these computational systems: Participants' current representations of their closest friends and their properties. For example, if participants report preferences for friends whom they perceive to give fewer benefits, be less powerful, or value others more than them, then the respective hypothesis is contradicted.

We tested whether people readily rank their close friends in a way that is meaningful to them. Alliance-building requires an egocentric friend-ranking. In contrast, Fiske [10] claimed that communal relationships observe a “principle of equivalence,” meaning that close friends should be undifferentiated and any ranking should be meaningless. Anecdotally, people frequently assert that they value all friends equally. However, this phenomenon might be a tactic for hiding friends' ranks (see above regarding the “straddle strategy”). To investigate this, we looked at whether people are motivated to conceal their friend-ranking in public.

Finally, and most centrally, we examined individuals' perceptions of their friends' properties to see which properties predict higher friend rank. We selected properties that fit with candidate explanations for friendship, including alliance-building and bandwagoning, as well as theories surrounding exchange [7], [35]–[37], familiarity [38], proximity [39], and similarity [40]. These theories imply different hypotheses about which variables will be the strongest predictors of friend rank. For example, exchange theories predict that received benefits should dominate, whereas familiarity predicts that frequency of contact should be most important.

## Methods

### Ethics Statement

Participants received a consent form which they were required to read. Written consent was not required because the data were analyzed anonymously. The consent form, consent procedure, and all study procedures were approved by the University of Pennsylvania Institutional Review Board.

### Study 1

#### Design

To examine friend-ranking, we used a point allocation task. Constant-sum allocation tasks have been used in marketing research to examine consumer preferences [41]. If ranking friends is not meaningful to participants (because friends are undifferentiated and valued equally), then point allocations should be uniformly distributed across friend-ranks. However, if people readily rank friends, then point allocations should be skewed, with high-ranking partners attracting a greater proportion of limited friendship points.

We examined whether people hide their rankings with a within-subject public manipulation. Participants repeated the allocation task while imagining that their friends would know their decisions. If participants conceal ranking, disparity in point allocations should shrink in public, with high-ranking friends receiving fewer points and low-ranking friends receiving more points, relative to the private condition.

Finally, the main task of this study was designed to investigate predictors of friend-rank. We used a repeated measures design in which we measured participants' friendship attributes for ten friends. Central to the alliance hypothesis, one of these attributes was the participant's own perceived rank among each partner's other friends. To look at exchange, we asked participants to rate the amount of material and social benefits that they receive as a result of the friendship. To the extent that friends function as trade partners, the volume of incoming benefits should influence closeness. Participants also rated similarity, secret sharing, several traits (caring, intelligence, attractiveness, and popularity), and they indicated duration, frequency of contact, sex, and age.

#### Participants, Materials, and Procedure

We recruited 54 participants (26 females, 28 males; ages 18–22 years, *M* = 18.94; *SD* = 1.12) to answer questions about ten of their friendships, providing information about 540 friendships. Participants were undergraduates at the University of Pennsylvania. After giving informed consent, participants completed a friendship questionnaire at the Penn Laboratory for Experimental Evolutionary Psychology. The questionnaire is included as Supporting Information S1. The questionnaire consisted of the following items.

#### Friend-ranking task

Participants listed the initials of their 10 best friends in rank order from best friend to tenth best friend. They were instructed to exclude family members and romantic/sexual partners.

#### Friendship point allocation task

Participants divided a limited budget of 100 points among their ten best friends in proportion to the participant's relative closeness with each friend. This task was completed in two within-subject conditions: a private condition and a public condition; in the latter, participants were instructed to imagine that their friends would know their allocation decisions. The instructions of the public task inherently draw attention to the private-public distinction, but this is not true of the private task. Hence, we administered the private condition before the public condition, without counterbalancing, to minimize any effects of knowing the manipulation on the private allocations. Whereas the nature of the public task creates the possibility of experimenter demand, we think it is unlikely that participants could infer our specific hypothesis (points distributed more equally) unless they shared the friendship intuitions here under study. Nonetheless, we examined order effects with a counterbalanced design in Study 3 (see below).

#### Friendship properties measures

Participants answered items about the properties of each friend or friendship. Central to our hypotheses, they reported their perceived rank in each partner's friend-ranking. Also, using a seven-point scale (1 = *low* and 7 = *high*), participants reported received benefits, similarity, and secret sharing. To examine friends' traits, participants used the same scale to rate each friend's caring, intelligence, attractiveness, and popularity. Finally, participants reported each friend's age and sex, friendship duration (years), and frequency of contact (per week).

Participants completed the items in this order: ranking task, the private allocation task, the properties measures, and then the public allocation task. Upon completion of the questionnaire, participants were debriefed and dismissed. The procedure lasted 25 minutes.

#### Data Analysis

We used ordinal logistic regression to examine predictors of friend-rank among our measures of friendship properties. Logistic regression fits a linear model to the logit (log odds) of a discrete dependent variable; the coefficient *β* is in terms of the log odds, and therefore, exp(*β*) gives the change in odds per unit change in the associated predictor. When the dependent variable has more than two categories, and the categories possess a natural order, ordinal logistic regression is appropriate. In this case, exp(*β*) describes the change in the generalized odds of being in a higher category in the ordinal scale. We used standardized values to compare coefficients across variables, so coefficients indicate the change in the odds for an increase of one standard deviation for a given independent variable. For example, *β* = 1.68 for perceived rank in Study 1 (see below) indicates that for each unit (*SD*) increase in perceived rank, friends have exp(1.68) = 5.37 times greater odds of being ranked a better friend. Finally, because the data are repeated measures (participants' responses for each of ten friends) and the present research focuses on individual level effects, we included subject as a random effect in the model.

### Study 2

Important changes occur in individuals' friendships during the transition from high school to college [42]. Thus, college undergraduates might differ from the broader population in their friendship patterns. Study 2 replicated Study 1 with participants recruited from Center City Philadelphia.

#### Participants, Materials, and Procedure

In Center City Philadelphia, 49 participants (22 females, 27 males) were paid $5 to complete friendship questionnaires. In the park at Rittenhouse Square, we approached individuals who were not engaged in social interaction. Participants answered questions about ten of their friendships, providing information about 490 friendships. The participants ranged in age from 18 to 65 years, *M* = 31.76, *SD* = 13.33. We used the same materials and procedure as in Study 1.

### Study 3

Study 3 replicated the previous studies in a larger web-based sample.

#### Participants, Materials, and Procedure

We recruited 182 (120 females, 62 males) participants to answer questions about ten of their friendships, providing information about 1,820 friendships. Participants volunteered to participate in an online study for which they received a small payment. Participants ranged in age from 18 to 70, *M* = 31.00, *SD* = 10.69. The materials and procedure were the same as in Study 1 except that the questions were converted to html format.

## Results

### Study 1

#### Point Allocations in Private and Public

**Figure 1:**
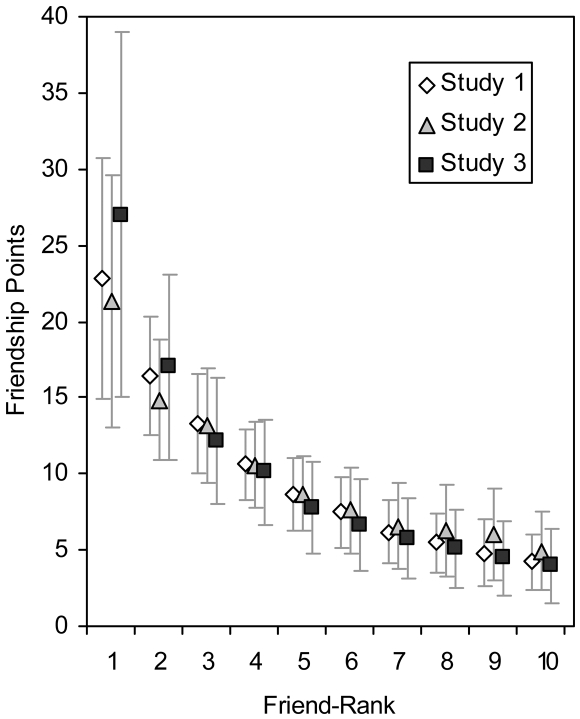
Mean (*SD*) point allocations by friend-rank. The null hypothesis that people do not rank their closest friends predicts a flat line at 10 points.

Point allocations dropped off steeply as a function of rank, with best friend attracting nearly a quarter of the total points (Figure 1). A chi-square goodness-of-fit test showed that the aggregate point allocations differed significantly from a uniform distribution, χ^2^ (9, *N* = 5,400) = 1,735.21, *p*<.001. Looking at just the top two friends, points allocated to the best friend differed from points allocated to the 2^nd^ best friend, χ^2^ (1, *N* = 2,121) = 56.04, *p*<.001.

**Figure 2:**
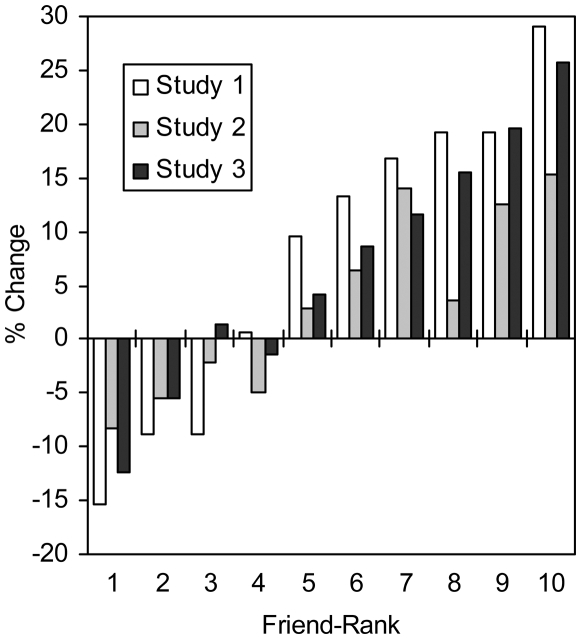
The change in aggregate point allocations caused by the public manipulation. Bar values represent the public minus private difference divided by private points to give the percent change.

The public manipulation changed aggregate point allocations (Figure 2). As hypothesized, high-ranking friends received fewer points in public, while low-ranking friends received more points. Looking at the individual level, we computed for each participant the average (absolute) allocation difference across the 45 pairings among their ten closest friends. That is, for each participant's set of 10 allocation amounts, *y*, we computed the mean difference (*MD*), given by:
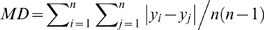



The *MD*, a common measure of statistical dispersion, was used to measure non-equivalence in friendship point allocations. Mean (*SD*) values for this metric were 6.8 (2.6) points in private and 5.3 (3.2) points in public. Looking within-subject, dispersion in public was less than, equal to, and greater than the private condition for 61%, 24%, and 14% of participants, respectively. Because the data violated normality, we conducted a Wilcoxon signed-rank test, finding that participants' allocations exhibited less dispersion in public than in private, *Z* = 4.41, *p*<.001, one-tailed.

#### Friendship Properties and Friend-Rank

Table 1 reports descriptive statistics for the friendship properties measures. We analyzed the relationships between these properties and friend-rank. For ease of interpretation, we transformed friend-rank and perceived rank by multiplying by -1 so that high-ranking individuals, such as friend number 1, are denoted by numerically greater values than low-ranking individuals, such as friend number 10. In a preliminary analysis, we computed simple correlations for each participant between rank and friend properties (treating rank as continuous). Table 2 reports the mean (*SD*) for participants' correlations between friend rank and each friendship property. The largest correlations were with perceived rank, secrets, similarity, and benefits.

**Table 1:** Descriptive Statistics for Friendship Properties.

Variable	Study 1		Study 2		Study 3		
	*M*	*SD*	*M*	*SD*	*M*	*SD*	Range^a^
Perceived rank	5.17	3.58	5.28	3.68	6.73	5.52	1–40
Benefits	3.74	1.78	4.31	1.89	3.52	1.90	1–7
Similarity	4.34	1.52	4.43	1.62	4.05	1.71	1–7
Frequency (per week)	6.84	8.67	4.71	7.41	4.58	6.39	0–35
Duration (yrs)	5.17	4.04	9.69	10.21	8.93	8.04	0–65
Secrets	4.27	1.82	4.80	1.86	4.05	2.07	1–7
Caring	5.22	1.35	5.34	1.59	5.15	1.62	1–7
Popularity	5.11	1.35	5.49	1.50	4.79	1.67	1–7
Intelligence	5.49	1.25	5.66	1.30	5.22	1.49	1–7
Attractiveness	4.85	1.39	5.32	1.38	4.68	1.56	1–7
Same-sex^b^	.78	.41	.69	.46	.74	.44	0–1
Age difference (yrs)	0.46	1.23	3.69	6.54	4.19	5.89	0–47

*Note.* Mean and standard deviation for properties of participants’ friendships.

a The range across all three studies.

b Same-sex = 1, opposite-sex = 0.

**Table 2:** Correlations with Friend-Rank.

Variable	Study 1^a^		Study 2^b^		Study 3^c^	
	*M*	*SD*	*M*	*SD*	*M*	*SD*
Perceived rank	.71	.25	.50	.38	.68	.34
Benefits	.45	.35	.45	.37	.45	.42
Similarity	.55	.32	.39	.37	.53	.37
Frequency (per week)	.16	.48	.24	.41	.40	.40
Duration (yrs)	.25	.41	.22	.34	.29	.44
Secrets	.60	.33	.40	.42	.64	.34
Caring	.30	.39	.25	.36	.33	.36
Popularity	.16	.34	.18	.35	.11	.42
Intelligence	.13	.37	.16	.31	.23	.35
Attractiveness	.03	.37	.13	.34	.15	.37

*Note.* Mean (*SD*) values across participants for correlations between each variable and friend-rank. Friend-rank and perceived rank variables were transformed by multiplying by -1.

a Study 1 means are significantly different (Bonferroni corrected) from zero except frequency, intelligence, and attractiveness.

b Study 2 means are significant except attractiveness.

c All Study 3 means are significant.

Because friend-rank is an ordinal variable, we constructed an ordinal logistic model of friend-rank (Table 3). The ordinal logistic regression allowed us to look at each predictor controlling for all other factors. The variables friend-rank, perceived rank, and age difference were multiplied by -1 for ease of comparison with the other variables. Predictors were first assessed for collinearity by examining intercorrelations; correlations were relatively small with the highest absolute value at *r* = .48. Because we were interested in individual level effects, we entered subject in the model as a random effect. The model overall was statistically significant, χ^2^(66) = 544.58, *p*<.001. Looking at the coefficients, the strongest significant predictors (in order of magnitude) were perceived rank, popularity, benefits, similarity, secrets, same-sex, attractiveness, and intelligence. The non-significant factors were frequency, duration, caring, and age difference.

**Table 3:** Ordinal Logistic Model of Friend-Rank

Variable	Study 1			Study 2			Study 3		
	*β* a	*SE*	Wald χ^2^	*β* a	*SE*	Wald χ^2^	*β* a	*SE*	Wald χ^2^
Perceived rank	1.68	0.14	156.95***	0.77	0.11	49.25***	1.21	0.08	238.83***
Benefits	0.55	0.11	22.41***	0.60	0.13	24.27***	0.48	0.06	56.30***
Similarity	0.49	0.12	17.19***	0.41	0.11	14.93***	0.70	0.06	128.52***
Frequency	−0.17	0.09	1.33	0.22	0.07	3.29	0.44	0.10	20.09***
Duration	0.16	0.12	2.15	0.20	0.10	2.97	0.21	0.06	13.08***
Secrets	0.55	0.13	17.06***	0.33	0.11	8.60**	0.77	0.07	128.13***
Caring	0.05	0.11	0.31	−0.02	0.11	0.02	0.09	0.06	2.34
Popularity	0.58	0.11	27.92***	0.12	0.11	1.43	0.09	0.06	3.05
Intelligence	0.21	0.10	4.51*	0.10	0.10	0.91	−0.02	0.06	0.09
Attractiveness	−0.25	0.10	5.58*	0.06	0.11	0.27	0.05	0.06	0.86
Same-sex	0.29	0.05	9.39**	0.21	0.05	5.70*	0.03	0.02	1.88
Age difference	0.02	0.09	0.07	0.07	0.13	0.10	0.27	0.05	24.06***

*Note.* Effect tests for ordinal logistic model of friend-rank. Friend-rank and perceived rank variables were transformed by multiplying by −1.

a Standardized logistic regression coefficient. The exponential of *β* is the change in the odds of being ranked a better friend for each unit (*SD*) increase in the associated predictor.

* *p*<.05. **p<.01. ***p<.001.

### Study 2

#### Point Allocations in Private and Public

Mean (*SD*) point allocations are shown in Figure 1. A chi-square goodness-of-fit test showed that the aggregate point allocations differed significantly from a uniform distribution, χ^2^(9, *N* = 4,900) = 1,159.03, *p*<.001. Looking at just the top two friends, points allocated to the best friend differed from points allocated to 2^nd^ best friend, χ^2^ (1, *N* = 1,772) = 56.35, *p*<.001.

Point allocations differed in the public condition relative to the private condition (Figure 2). To examine changes at the individual level, we compared the mean difference (*MD*) of participants' allocation values in private and public conditions. Mean (*SD*) values for this measure were 6.4 (2.0) points in private and 5.5 (3.4) points in public. In the public condition, dispersion was less than, equal to, and greater than the private condition for 39%, 43%, and 18% of participants, respectively. A Wilcoxon signed-rank test showed that participants' allocations exhibited less dispersion in public than in private, *Z* = 2.24, *p* = .02, one-tailed.

#### Friendship Properties and Friend-Rank

Table 1 reports descriptive statistics for the friendship properties measures. Compared with the Penn students in Study 1, participants reported longer friendship durations (*M* = 9.69 versus 5.17 years) and greater absolute age differences (*M* = 3.69 versus 0.46). Table 2 reports the mean (*SD*) for participants' correlations between friend rank and each friendship property. The largest correlations were with perceived rank, benefits, secrets, and similarity.

We conducted an ordinal logistic regression to examine predictors of friend-rank (Table 3). As in Study 1, friend-rank, perceived rank, and absolute age difference were transformed by multiplying by -1. Predictors were assessed for collinearity by examining intercorrelations, which were relatively small with the highest absolute value at *r* = .46. Because we were interested in individual level effects, we entered subject in the model as a random effect. The model overall was statistically significant, χ^2^(61) = 244.11, *p*<.001. Looking at the coefficients, the strongest significant predictors (in order of magnitude) were perceived rank, benefits, similarity, secrets, and same-sex. The non-significant factors were frequency, duration, caring, popularity, intelligence, attractiveness, and age difference.

### Study 3

#### Point Allocations in Private and Public

Figure 1 shows the mean (*SD*) point allocations across partners with each friend-rank. A chi-square goodness-of-fit test showed that the aggregate point allocations differed significantly from a uniform distribution, χ^2^ (9, *N* = 18,200) = 8,426.58, *p*<.001. Looking at just the top two friends, points allocated to the best friend differed from points allocated to 2^nd^ best friend, χ^2^ (1, *N* = 8,009) = 415.00, *p*<.001.

Point allocations differed in the public condition, relative to the private condition (Figure 2). To examine changes at the individual level, we compared the mean difference (*MD*) of participants' allocation values in private and public conditions. Mean (*SD*) values for this measure were 7.86 (3.30) points in private and 6.67 (3.93) points in public. In the public condition, dispersion was less than, equal to, and greater than the private condition for 40%, 51%, and 10% of participants, respectively. A Wilcoxon signed-rank test showed that participants' allocations exhibited less dispersion in public than in private, *Z* = 6.00, *p*<.001, one-tailed.

In this and previous studies, participants completed the private allocation before the public allocation. To check for order effects, we ran an additional web-based sample of participants (*n* = 101; 61% female; age: *M* = 30.13, *SD* = 10.75) in the opposite order with the public allocation occurring before the private allocation. The mean (*SD*) values for the dispersion measure were 8.94 (4.09) points in private and 7.76 (3.80) points in public. In the public condition, dispersion was less than, equal to, and greater than the private condition for 46%, 41%, and 14% of participants, respectively. A Wilcoxon signed-rank test showed that participants' allocations exhibited less dispersion in public than in private, *Z* = 4.70, *p*<.001, one-tailed.

#### Friendship Properties and Friend-Rank

Table 1 reports descriptive statistics for the friendship properties measures. Friendship durations (*M* = 8.93) and absolute age differences (*M* = 4.19) were greater than for Study 1 and comparable to Study 2. Table 2 reports the mean (*SD*) for participants' correlations between friend rank and each friendship property. The largest correlations were with perceived rank, secrets, benefits, and similarity.

We conducted an ordinal logistic regression to examine predictors of friend-rank (Table 3). As in Study 1, friend-rank, perceived rank, and absolute age difference were transformed by multiplying by -1. Predictors were assessed for collinearity by examining intercorrelations, which were relatively small with the highest absolute value at *r* = .53. Because we were interested in individual level effects, we entered subject in the model as a random effect. The model overall was statistically significant, χ^2^(194) = 1,613.35, *p*<.001. Looking at the coefficients, the strongest significant predictors (in order of magnitude) were perceived rank, secrets, similarity, benefits, frequency, duration, and age difference. The non-significant factors were same-sex, caring, popularity, intelligence, and attractiveness.

## Discussion

People differentiated among their friends when allocating a limited budget of friendship points. In contrast to the idea that friends are equivalent and undifferentiated [10], people seemed to readily rank their friends. In fact, the greatest disparities were observed among individuals' closest friends (see Figure 1). This finding is consistent with an alliance-building model as well as theories surrounding exchange, similarity, and familiarity. Further, the effect of the modest manipulation–simply imagining that allocations would be known–suggests that people try to hide differences between friends. Perhaps the “principle of equivalence” observed for communal relationships reflects similar attempts to conceal sensitive alliance information.

Our main findings surrounded the predictors of participants' friend rankings. Consistent with exchange and similarity theories, we found significant effects for benefits and similarity in all three studies. Additionally, we found a striking effect for the key alliance variable: Individuals' own perceived rank was the strongest predictor of friend-rank. Perceived rank remained a powerful predictor after controlling for a range of variables from current theories of friendship. Friends' traits (e.g., intelligence, caring), and features identified in the friendship literature as important (similarity, benefits, frequency of contact, etc., [43]), were relatively weak predictors by comparison.

This finding is consistent with game theoretic analyses showing that individuals' traits have little influence over which alliances are formed [26], [31]. When individuals value each other precisely because their partner values them (versus the partner's traits), a self-reinforcing process of alliance-building is set in motion. Snyder [31] referred to this alliance dynamic as an “integrative spiral” and the effect can also be understood as a special case of the general idea proposed by Tooby and Cosmides [12] that friendship is the product of a self-reinforcing process.

Previous studies similarly found that friend liking was correlated with how much friends like oneself more than other friends [44], [45]. This pattern was interpreted as the result of preferences for absolute metrics such as received benefits, frequency of contact, or similarity [44]. The present findings challenge the idea that absolute metrics drive friendship choices by showing that a *relative* metric, one's perceived rank, remains a strong predictor of friend-rank after controlling for benefits, frequency of contact, and similarity.

It seems likely that participants' perceptions of their own rank position were often inaccurate (see [44]). Indeed, given our evidence that people conceal friend-ranking, ascertaining accurate rank information is probably difficult. We emphasize that models of friendship decisions do not make predictions about the real world of alliances, exchange, etc., but only about individuals' representations of the world. The underlying systems should aim to extract the relevant information, but when these evaluations fail, actual performance will only imperfectly approach system targets.

An important limitation of the present studies is that we did not directly manipulate perceived rank but relied on within-subject variation in participants' perceptions about their friends. Future work should aim to manipulate participants' perceptions of their friends' rankings, though this might be difficult and ethically questionable. However, it is possible that newly formed relationships in the laboratory exhibit patterns similar to long-term relationships, and if so, then it should be feasible to manipulate new friendships to better understand alliance-building decision processes.

Another limitation concerns the resolution of our instruments for measuring properties of friendships. Variables such as similarity and benefits are potentially vague and participants could have differed in their interpretations of these items. For instance, participants might have responded to the benefits item by focusing on past, current, or expected future benefits. For the exchange theory, the most relevant figures are the present values of the streams of benefits associated with each friend, including the discounted expected future benefits. It might be possible to assess participants' perceptions more precisely with higher resolution measures.

In general, our results suggest that human friend cognition might function, in part, to secure alliances. If so, economic approaches [7], [35]–[37] will be insufficient to capture this aspect of human friendship. Instead, the underlying psychology is likely to be strategically rich, sufficiently so to navigate the intricate network of existing and potential alliances [13], [14]. An analogy with international relations is informative. Nations look for different qualities in trade partners versus allies. The value of a trade partner depends on the potential for gains in exchange. In contrast, the value of an ally depends on the probability of support in possible future conflicts, which necessarily depends on the ally's commitments to others (and less on other traits). If political scientists tried to predict alliances on the basis of economic exchange or proximity, they would be disappointed. The United States and Mexico share close proximity and have important economic ties (e.g., NAFTA), but Mexico is by no means America's “best friend.” Similarly, in 2006 the United States traded with China over three times more than the U.K. (see www.census.gov), but China is hardly a better ally than the U.K. If human friendships are like international alliances, then friendship will not be well-explained by exchanges of benefits.

In conclusion, we find that human friend cognition shows patterns consistent with an alliance-building function. The alliance hypothesis offers an additional way to conceptualize and investigate human friendship. First, the alliance model draws attention to people's assessments of relative metrics rather than absolutes. For instance, alliance dynamics might help to explain why people are extremely concerned with social comparisons [46] or with others' relative superiority in knowledge or skills [47]. Psychological systems might be attuned to relatives, rather than absolutes, because others' alliance decisions are inherently comparative. Next, the alliance hypothesis might help explain jealousy and relational aggression among friends [48]. These phenomena seem inconsistent with both reciprocity theories and ideas about communal relationships. For example, models of indirect reciprocity [49] predict altruism toward those who help others–the opposite of jealousy toward friends' friends. In contrast, alliance problems provide an obvious motive for sabotaging others' cooperative relationships. Finally, if friends function as allies, then possible mechanisms can be drawn from existing theory in international relations, since nations have had to solve exactly the same problem–assembling a network of friends who provide support in conflicts. Psychology can leverage these tools from game theory to guide a new approach to understanding human friendship.

## Supporting Information

Supporting Information S1 Friendship Study Questionnaire
(0.02 MB PDF)
