# Supplementary material for: The Alliance Hypothesis for Human Friendship
Source: PLoS One. 2009 Jun 3;4(6):e5802. doi: 10.1371/journal.pone.0005802 (PMC2688027; doi:10.1371/journal.pone.0005802)
Supplement: Supporting Information S1 — Friendship Study Questionnaire (0.02 MB PDF) [file pone.0005802.s001.pdf]

Friend Survey

Please answer the following questions in the tables provided. Complete all items in the order they are presented. Do not return to previous questions or change earlier responses once you have moved on to subsequent sections.

I. Best Friends

Use the table on the next page to respond to the following.

Friends' Initials
Think of your 10 best friends. ***Do not*** *include current romantic/sexual partners or family members*. Please list your friends' initials in order of closeness, with your best friend listed first, your second best friend listed second, and so on. **Please repeat this exact list in all subsequent columns labeled "Friends' Initials"**.

Points Allocation
You have a budget of 100 points to distribute across your friends. Points indicate the closeness of your relationship with each friend. For example, if friend A has twice as many points as friend B, this indicates that you are twice as close to A as you are to B. Please use the scratch paper provided to ensure that the points in this column sum to 100.

Benefits
Consider the direct benefits that you receive as a result of this friendship. This includes material benefits such as favors or gifts, as well as social networking benefits. (This ***does not*** include psychological or emotional benefits.) Rate the total benefits on the following scale:

Low		Medium			High	
1	2	3	4	5	6	7

Similarity
Consider your overall similarity to this friend. Rate your overall similarity to this friend using the following scale:

Low		Medium			High	
1	2	3	4	5	6	7

	**Friends' Initials**	**Points Allocation** (Must add to 100)	**Benefits** (1-7)	**Similarity** (1-7)
**closest**	**1.**			
	**2.**			
	**3.**			
	**4.**			
	**5.**			
	**6.**			
	**7.**			
	**8.**			
	**9.**			
**least close**	**10.**			

II. Friend Information

Friendship Duration
Indicate how many years you have known this friend.

Your Priority
Imagine that this friend listed their best friends in order of closeness. Estimate what number you would be on their list of friends. For example, "1" indicates that they would list you as their best friend, while "15" indicates that you are their 15th best friend.

Secrets
Please rate the extent to which you tell this friend your secrets. Use the following scale:

Low		Medium				High
1	2	3	4	5	6	7

	Friends' Initials	Duration (years)	Your Priority	Secrets (1-7)
closest	1.			
↓	2.			
	3.			
	4.			
	5.			
	6.			
	7.			
	8.			
	9.			
least close	10.			

III. Character Traits

Please rate the following character traits in your friends and yourself. Use the following seven point scale:

Low		Medium				High
1	2	3	4	5	6	7

	Friends' Initials	Caring (1-7)	Intelligent (1-7)	Attractive (1-7)	Popular (1-7)
closest	1.				
↓	2.				
	3.				
	4.				
	5.				
	6.				
	7.				
	8.				
	9.				
least close	10.				

Yourself				

IV. Demographics

How Often
How frequently are you in contact with this friend each week (whether in passing, class, phone call, email, etc.)? *Please indicate the estimated average number of times you are in contact with your friend each week.*

	Friends' Initials	Sex (m/f)	Age	Ethnicity	How Often (# per week)
closest	1.				
	2.				
	3.				
	4.				
	5.				
	6.				
	7.				
	8.				
	9.				
least close	10.				

**Yourself**			

V. Public Information

In this section, please *<u>imagine that your friends will see your responses</u>*. For example, imagine that this table will be placed in a public place, such as the Internet. Please complete the items as in Section I, as though you expect your friends to see your responses.

	**Friends' Initials**	**Points Allocation** (Must add to 100)
**closest**	**1.**	
	**2.**	
	**3.**	
	**4.**	
	**5.**	
	**6.**	
	**7.**	
	**8.**	
	**9.**	
**least close**	**10.**	
